# Improvements in body composition and postural control in children with obesity following an AI-assisted, deficit-oriented exercise intervention

**DOI:** 10.3389/fpubh.2026.1886988

**Published:** 2026-09-17

**Authors:** Lixin Cao, Xingxing Cheng, Changqing Deng, Yuexin Ren, Shunjiu Zhang, Lunyu Li

**Affiliations:** School of Sport Medicine and Health, Chengdu Sport University, Jianyang, Sichuan Province, China

**Keywords:** AI-assisted decision support system, childhood obesity, deficit-oriented, postural control, precision exercise intervention

## Abstract

**Objective:**

To investigate the effects of an AI-assisted, individualized exercise management model on body composition, physical fitness, and postural stability in children with obesity, providing a feasibility foundation for subsequent randomized controlled trials.

**Methods:**

A single-group, pre-post pilot study was conducted. Nineteen children with simple obesity (7 girls, 12 boys; mean age 10.11 ± 2.0 years) from West China Second Hospital (July–September 2024) completed a 12-week exercise intervention. The AI system generated individualized FITT-VP prescriptions based on baseline assessments and dynamically adjusted them via training feedback, with on-site coach implementation. Outcomes were measured pre- and post-intervention.

**Results:**

All 19 children completed the intervention with no adverse events. Primary outcomes: BMI decreased from 25.29 ± 3.56 to 23.64 ± 3.60 kg/m^2^ (*p* < 0.001, *d* = 0.46), and body fat mass from 21.06 ± 6.70 to 17.94 ± 6.69 kg (*p* < 0.001, *d* = 0.47). Secondary outcomes: waist circumference (*p* < 0.01, *d* = 0.31), grip strength (*p* < 0.01, *d* = 0.54), 1-min jump rope (*p* < 0.01, *d* = 1.15), 1-min sit-ups (*p* < 0.05, *d* = 1.00), and sit-and-reach (*p* < 0.05, *d* = 0.67) all improved significantly. In the eight-directional postural control assessment, endpoint excursion and maximum excursion exhibited improvement trends in all directions, and reaction time was significantly reduced in four directions (*p* < 0.05). In the Sensory Organization Test, the mean balance percentages for SOT-4 and SOT-5 significantly increased (*p* < 0.01, *d* = 1.05 and *d* = 0.58, respectively), and the composite balance score increased by 24.15% (*p* < 0.001, *d* = 1.28). The visual ratio significantly increased by 19.5% (*p* < 0.05, *d* = 0.80), whereas the vestibular ratio increased by 15.3%, approaching but not reaching statistical significance (*p* = 0.054, *d* = 0.43).

**Conclusion:**

The AI-assisted, deficit-oriented exercise intervention demonstrated good feasibility and safety in this cohort of children with obesity. The observed improvements in body composition, motor function, and postural control indicators provide a feasibility basis for subsequent confirmatory trials on precision exercise management for childhood obesity.

## 1 Introduction

The prevalence of childhood obesity continues to rise and is recognized as a major global health concern (1). In China, the overall prevalence of overweight and obesity among children and adolescents aged 6 to 17 years has reached 19.0%, with a trend toward younger age groups (2). Obesity not only increases the risks of insulin resistance (3), thyroid dysfunction (4), and psychosocial maladjustment (5) during childhood but is also closely associated with cardiovascular disease in adulthood (6).

Notably, children with obesity frequently exhibit impairments in postural control and balance due to mechanical, sensory, and neuromuscular factors. These deficits may lead to delayed motor development, reduced mobility, and difficulties in motor skill acquisition (7–9). Previous studies have demonstrated that obese children tend to adopt a more anteriorly shifted center-of-gravity strategy during physical activities (10). This forward-shifted strategy significantly increases the risk of falls and sports injuries and limits participation in activities requiring rapid directional changes and dynamic balance. This creates a vicious cycle of “obesity → poor postural control → reduced physical activity → low energy expenditure → progressive obesity,” underscoring the urgent need for early postural control intervention.

Traditional exercise intervention models, however, generally adopt a “one-size-fits-all” approach (11), which fails to address individual differences among children. Furthermore, existing protocols exhibit considerable heterogeneity across studies, leading to inconsistent clinical practices (12). In addition, remote guidance models relying on mobile applications often yield limited adherence and effectiveness due to the absence of external supervision and real-time feedback (13).

To address these limitations, the present study developed a deficit-oriented exercise intervention model that integrates AI-assisted decision-making with on-site coach implementation. The novelty of this model is threefold. First, it adopts an intervention philosophy that targets individual functional deficits. Based on eight-directional postural control assessment results, the system automatically identifies weak directions and assigns greater training weight to the relevant muscle groups, achieving precise strengthening according to the principle of “training where deficits exist.” Second, unlike most remote guidance models, this study employs a closed-loop hybrid framework in which the AI system is responsible for data-driven prescription optimization, while the coach provides on-site supervision, movement correction, and motivational support. This framework balances decision accuracy with implementation adherence. Third, whereas previous studies have primarily focused on changes in body composition, the present study systematically incorporates eight-directional postural control and sensory integration tests to comprehensively evaluate the intervention’s effects at the neuromuscular level. Based on this rationale, this study aimed to investigate the effects of this AI-assisted, deficit-oriented exercise intervention on body composition, motor performance, and postural control in children with obesity, thereby offering a functional medicine perspective and practical foundation for precision exercise management in this population.

## 2 Subjects and methods

### 2.1 Subjects

A total of 19 eligible children with obesity (7 girls, 12 boys; mean age 10.11 ± 2.0 years) who visited the Department of Child Health at West China Second University Hospital, Sichuan University, between July and September 2024 were enrolled. Written informed consent was obtained from their legal guardians. Medical histories were collected, and physical examinations and laboratory tests were performed. Inclusion criteria were as follows: (1) meeting the obesity criteria defined by the National Health and Family Planning Commission standard WS/T 586–2018, “Screening for Overweight and Obesity in School-Aged Children and Adolescents,” i.e., BMI exceeding the 95th percentile for age and sex (14); (2) no contraindications to exercise; (3) no participation in systematic weight-loss or exercise interventions within the preceding 3 months. Exclusion criteria were as follows: (1) pathological obesity (e.g., hypothyroidism, Cushing’s syndrome); (2) severe dysfunction of vital organs (heart, lungs, liver, or kidneys); (3) skeletal deformities, muscle disorders, or neurodevelopmental conditions that precluded completion of standardized exercise; (4) use of medications affecting body weight or metabolism (e.g., glucocorticoids, growth hormone) within the preceding 3 months; (5) inability to comply with data submission requirements, voluntary withdrawal, or missing critical data during the intervention period. This study was approved by the Medical Ethics Committee of West China Second University Hospital, Sichuan University (approval number: 2023–142) and was registered with the Chinese Clinical Trial Registry (ChiCTR2500099563).

### 2.2 Sample size estimation

This study was designed as a single-arm pre-post pilot trial. According to methodological guidelines, the sample size for a pilot study should be determined based on feasibility objectives rather than hypothesis-testing power calculations (15). Julious (16) proposed that a minimum of 12 participants per group was recommended for pilot studies without prior data. Considering the single-arm design and an anticipated dropout rate of 15 to 20%, a total of 19 participants were enrolled. This sample size is consistent with recommended guidelines and was deemed sufficient to evaluate trial feasibility and intervention effects.

### 2.3 Intervention protocol

The “Guidelines for Assessing Physical Activity Levels in Children and Adolescents Aged 7–18,” issued by the National Center for Disease Control and Prevention, recommends that children and adolescents engage in at least 70 min of moderate-to-vigorous physical activity daily (17). Accordingly, the intervention in this study consisted of three 90-min sessions per week, balancing intervention dose with practical feasibility for children. A hybrid model combining “AI-generated prescriptions” with “coach-led implementation” was adopted.

#### 2.3.1 Prescription generation logic of the AI-assisted exercise decision support system

The AI-assisted exercise decision support system used in this study is essentially an expert knowledge-driven system built upon a sports medicine rule base. This rule base integrates information from Exercise Prescription (18), the ACSM Guidelines for Exercise Testing and Prescription (19), the Guidelines for Physical Activity in Children and Adolescents Based on the Integration of Physical Education and Health (2024) (20), and the practical experience of sports medicine specialists. The internationally recognized FITT-VP framework (Frequency, Intensity, Time, Type, Volume, and Progression) serves as the core decision-making structure. The system automatically retrieves baseline data for each participant, including age, sex, height, weight, BMI, body fat mass, and skeletal muscle mass, as well as physical fitness indicators (grip strength, 1-min jump rope, 1-min sit-ups, and sit-and-reach) and eight-directional postural control data. By integrating individual obesity characteristics and functional deficits, the system performs multidimensional parameter matching based on FITT-VP principles to automatically generate a personalized exercise prescription tailored to the child’s physiological profile, fitness level, and specific postural control weaknesses. This approach enables intelligent and precise decision-making regarding exercise intensity, duration, type, total volume, and progression pace.

#### 2.3.2 Baseline dose setting

Aerobic exercise: frequency, 3 times per week; duration, 40–50 min per session; intensity, target heart rate = resting heart rate + (220 − age − resting heart rate) × 60–70%, corresponding to an RPE of 12–14. Resistance training: frequency, 3 times per week; duration, 20–25 min per session; intensity, 8–12 repetitions per set, 2–3 sets, corresponding to an RPE of 15–17. Aerobic exercises included brisk walking, jogging, jump rope, and aerobic dance. Resistance exercises included glute bridges, Romanian deadlifts, eccentric heel raises, planks, and resistance band rows.

#### 2.3.3 Identification of functional deficits and threshold determination

The identification of “functional weakness” was based on an empirical cutoff point (ECP): when the endpoint excursion (EPE) or maximum excursion (MXE) in a given direction fell below 30% of the age- and sex-specific reference mean, that direction was classified as functionally deficient. This ECP was selected based on the balance between standard error of measurement (SEM) and minimum detectable change (MDC) in clinical metrology. Previous biomechanical pilot work by our team estimated the SEM of this test in children to be approximately 12–15%. Setting the threshold too low (e.g., 20%) would result in excessive overlap with the 1.96× $\sqrt{2}$ ×SEM error interval, increasing the risk of false-positive classifications (Type I error) due to system noise or spontaneous sway. Conversely, setting it too high (e.g., 40%) would improve specificity but could miss clinically meaningful mild or early-stage deficits (Type II error). Therefore, the 30% threshold was chosen as the optimal decision boundary for balancing measurement error mitigation and clinical sensitivity.

#### 2.3.4 Personalized weight allocation

Based on the spatial distribution of identified weaknesses, the training load (number of sets) for exercises targeting the relevant muscle groups (erector spinae, gluteus maximus, hamstrings, and triceps surae) was automatically increased by the system. For instance, when posterior (B) EPE was weak, the set volume for eccentric control exercises targeting the posterior chain was increased by 50%; when posterolateral (LB, RB) directions were weak, the training load for lateral stabilizing muscles (gluteus medius and quadratus lumborum) was increased accordingly. Furthermore, the weak direction was integrated into the postural control-specific training component of each session, and the number of repetitions performed in that direction was doubled.

#### 2.3.5 Dynamic adjustment, coach discretion, and deviation management

Using the coach’s weekly feedback on movement quality (scored 1–5) and subjective fatigue (RPE 6–20), the system automatically adjusted training intensity for the subsequent week. If a child successfully completed the current number of sets/repetitions with an RPE ≤ 14 on two consecutive occasions, the number of sets was increased by 10%, or the inter-set rest interval was shortened by 5 s the subsequent week. The coach’s discretionary authority was limited to movement correction, safety protection, RPE inquiry, and emotional reassurance; the coach was not permitted to modify core prescription parameters (intensity, sets, or exercise type). Regarding deviation management, if a child failed to complete 80% of the prescribed training volume in two consecutive sessions, the system suspended the current prescription and required the child to return for a repeat eight-directional postural control assessment, after which the investigator determined whether a new prescription was needed.

### 2.4 Outcome measures

#### 2.4.1 Primary outcome measures

To control the Type I error rate, BMI and body fat mass were prespecified as the primary outcome measures. All other measures, including waist circumference, skeletal muscle mass, motor function tests, eight-directional postural control indicators, and SOT parameters, were defined as secondary outcomes.

#### 2.4.2 Anthropometric and body composition measurements

Body weight, BMI, waist circumference, waist-to-height ratio, body fat mass, and skeletal muscle mass were measured using an InBody body composition analyzer. Measurements were obtained with children fasting and after bladder emptying. Waist circumference was measured at the midpoint between the mid-axillary line and the anterior superior iliac spine, and the mean of three consecutive measurements was recorded. BMI was calculated as weight (kg) divided by height squared (m^2^). The waist-to-height ratio was calculated as waist circumference (cm) divided by height (cm). Measurements were taken before and after the 12-week intervention.

#### 2.4.3 Motor function tests

Before and after the intervention, the following assessments were conducted by a certified therapist: (1) grip strength: measured three times on the dominant hand in a standing position using a Jamar dynamometer, with the maximum value recorded; (2) 1-min jump rope and 1-min sit-ups: performed according to the National Student Physical Fitness Standards (21); (3) sit-and-reach: measured using a sit-and-reach tester, with the maximum value of two attempts recorded.

#### 2.4.4 Postural control assessment

Postural control was assessed using the NeuroCom Balance Manager 9.0 system, including the Limits of Stability (LOS) test and the Sensory Organization Test (SOT). Children stood barefoot on the force plate with both hands placed above the iliac crests.

#### 2.4.5 Limits of stability (LOS) test

This test assesses children’s ability to voluntarily shift their center of gravity in eight directions (forward, F; left-forward, LF; left, L; left-backward, LB; backward, B; right-backward, RB; right, R; right-forward, RF) while maintaining postural stability. (Figure 1).

**Figure 1:**
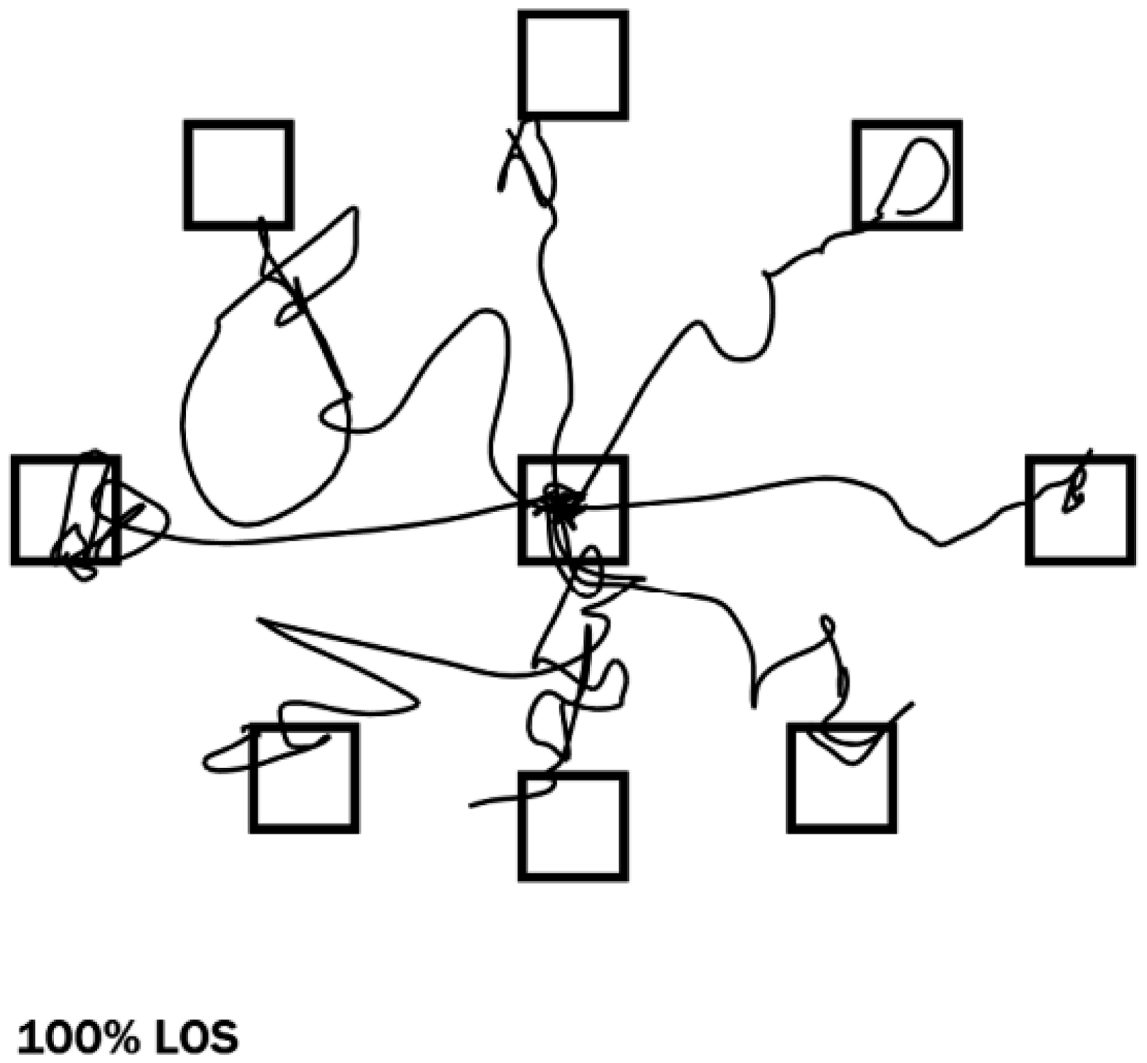
Schematic diagram of the eight-direction limits of stability (LOS) test.

Children observed the real-time projection of their center of gravity on the screen. They were instructed: “When you hear the prompt and see the target circle, shift your center of gravity to move the cursor to the target as quickly and accurately as possible, and maintain that position until the second prompt.” Children were allowed to use any movement strategy, provided they did not move or lift their feet. All trials began from the central square, with the first target in the forward direction, followed by clockwise sequencing. The following parameters were recorded: reaction time (RT), movement velocity (MVL), directional control (DCL), maximum excursion (MXE), and endpoint excursion (EPE) (Table 1). Two trials were performed for each direction, and the average of the two trials was used as the final score.

**Table 1:** Definitions of measurements from the NeuroCom® VSR Sport.

Measure	Definition	Unit
Reaction time	Time from the cue to the time the COP sway exceeds the random range indicating volitional movement has begun	Seconds (s)
Movement velocity	Average speed of the COG shift toward the target measured in degrees per second	Degrees per second (°/s)
Endpoint excursion	Distance traveled by the COG on the primary attempt to reach the target expressed as a percentage of the LOS	Percentage of LOS (%LOS)
Maximum excursion	Furthest distance traveled by the COG in a given trial	Percentage of LOS (%LOS)
Directional control	Amount of movement in the intended direction minus the amount of off-axis movement given as a percentage	Percentage (%)

COP, center of pressure; COG, center of gravity; LOS, limits of stability.

#### 2.4.6 Sensory organization test (SOT)

The SOT identifies the influence of different sensory inputs on postural balance. Under six conditions, the child’s ability to maintain balance and the motor strategies used were assessed by suppressing unreliable visual, vestibular, or proprioceptive information. The six conditions were as follows: condition 1 (eyes open, fixed platform, fixed visual surround); condition 2 (eyes closed, fixed platform); condition 3 (eyes open, sway-referenced visual surround, fixed platform); condition 4 (eyes open, sway-referenced platform, fixed visual surround); condition 5 (eyes closed, sway-referenced platform); condition 6 (eyes open, sway-referenced visual surround and platform) (Figure 2). Three 20-s trials were conducted for each condition.

**Figure 2:**
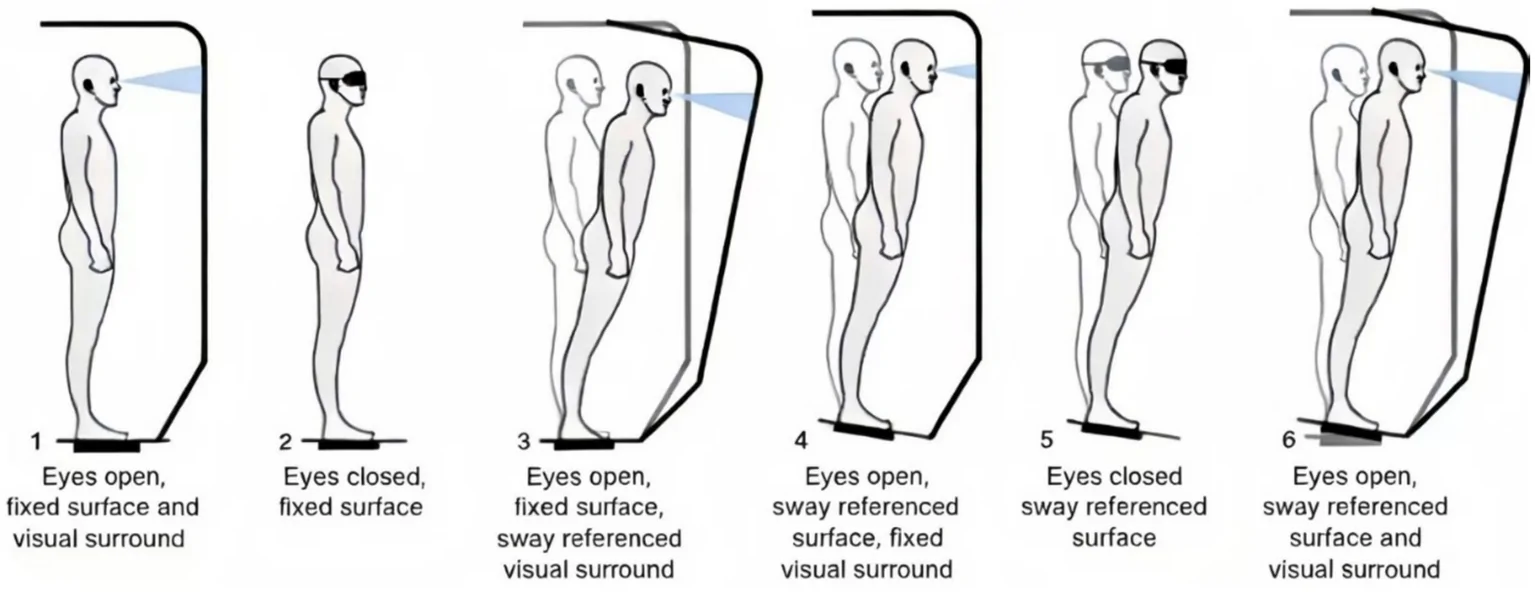
Six measurement conditions of the sensory organization test (SOT).

Four quantitative indices reflecting postural regulation, including the composite equilibrium score alongside somatosensory, visual, and vestibular sensory ratios, were calculated from equilibrium values acquired under all six SOT testing conditions.

#### 2.4.7 Diet and physical activity records

This study did not systematically monitor daily dietary intake, physical activity, sleep duration, screen time, family support, or motivation. During the study period, families were verbally instructed to “maintain their usual dietary habits as much as possible and avoid intentional dieting or overeating” to minimize dietary confounding. However, these verbal instructions could not replace objective dietary records; therefore, the possibility that some families may have consciously or unconsciously altered their dietary habits due to participation cannot be excluded, which may have influenced body composition changes.

### 2.5 Statistical methods

Statistical analyses were performed using SPSS Statistics version 27.0. Continuous variables were tested for normality using the Shapiro–Wilk test. Data following a normal distribution were expressed as mean ± standard deviation (SD). Paired *t*-tests were used for within-group comparisons before and after intervention. Statement on multiple comparisons: As this was a single-arm pilot study with the primary objective of exploring improvement signals and providing feasibility data for subsequent confirmatory trials (22), no correction for multiple comparisons (e.g., Bonferroni) was applied to the paired t-tests for secondary outcomes. This decision was made to avoid increasing the risk of Type II errors (false negatives) due to overcorrection. Therefore, all *p*-values for secondary outcomes should be interpreted as descriptive statistics, and the findings should be regarded as exploratory, requiring confirmation in future confirmatory trials. Effect sizes were calculated using Cohen’s *d* (0.2, 0.5, and 0.8 representing small, medium, and large effects, respectively). Coefficients of variation (%CV = SD/mean × 100%) were calculated for pre-intervention measures. A *p* < 0.05 was considered statistically significant.

## 3 Results

### 3.1 Participant adherence and safety

All 19 children completed the full 12-week intervention (36 sessions), with an overall attendance rate of 100%. No exercise-related muscle strains, joint injuries, or cardiovascular adverse events occurred during the intervention, indicating that the AI-assisted, deficit-oriented exercise intervention was both safe and feasible.

### 3.2 Changes in anthropometric and body composition parameters

After 12 weeks of intervention, body composition and anthropometric parameters showed significant improvements (Table 2). For the primary outcome measures, BMI decreased from 25.29 ± 3.56 kg/m^2^ to 23.64 ± 3.60 kg/m^2^ (*p* < 0.001, Cohen’s *d* = 0.46, small effect), and body fat mass decreased from 21.06 ± 6.70 kg to 17.94 ± 6.69 kg (*p* < 0.001, Cohen’s *d* = 0.47, small effect). For the secondary outcome measures, waist circumference decreased from 79.70 ± 9.19 cm to 76.71 ± 10.39 cm (*p* < 0.01, Cohen’s *d* = 0.31, small effect); waist-to-height ratio decreased from 0.542 ± 0.037 to 0.513 ± 0.046 (*p* < 0.001, Cohen’s *d* = 0.70, medium effect); and triceps skinfold thickness significantly decreased (*p* < 0.01, Cohen’s *d* = 0.83, large effect). Skeletal muscle mass showed no significant difference before and after intervention (*p* = 0.372), suggesting that lean mass was preserved while fat mass was reduced.

**Table 2:** Comparison of anthropometric and body composition indicators before and after intervention (mean ± SD, *n* = 19).

Indicator (unit)	Pre-intervention	Post-intervention	Mean difference	SD	*t*-value	*p*-value	CV%	Cohen’s *d*	Effect size
Height (cm)	146.968 ± 13.574	148.921 ± 12.936	−1.953	1.102	−7.726	*p* < 0.001	9.24%	−0.147	Negligible
Body weight (kg)	56.139 ± 16.858	53.642 ± 16.047	2.497	2.763	3.940	P < 0.001	30.03%	0.152	Negligible
BMI (kg/m^2^)	25.288 ± 3.556	23.643 ± 3.599	1.646	1.150	6.238	P < 0.001	14.06%	0.460	Small
Waist circumference (cm)	79.695 ± 9.187	76.705 ± 10.391	2.989	4.044	3.222	*p* < 0.01	11.53%	0.305	Small
Waist-to-height ratio	0.542 ± 0.037	0.513 ± 0.046	0.029	0.026	4.773	*p* < 0.001	6.83%	0.697	Medium
Triceps skinfold thickness (mm)	25.526 ± 4.303	22.037 ± 4.150	3.489	4.555	3.339	*p* < 0.01	16.86%	0.826	Large
Skeletal muscle mass (kg)	18.958 ± 6.674	19.221 ± 6.401	−0.263	1.254	−0.915	0.372	35.22%	−0.040	Trivial
Body fat mass (kg)	21.063 ± 6.703	17.937 ± 6.693	3.126	2.340	5.824	*p* < 0.001	31.82%	0.467	Small

### 3.3 Changes in motor function indicators

Multiple motor function indicators showed significant improvements (Table 3). Grip strength increased by approximately 14.9% (*p* < 0.01, Cohen’s *d* = 0.54, medium effect); 1-min jump rope performance improved by 35.3% (*p* < 0.01, Cohen’s *d* = 1.15, large effect); 1-min sit-ups increased by 36.4% (*p* < 0.05, Cohen’s *d* = 1.00, large effect); and sit-and-reach improved by 36.6% (*p* < 0.05, Cohen’s *d* = 0.67, medium effect). These results suggest that the intervention was associated with improvements in strength, endurance, coordination, and flexibility.

**Table 3:** Comparison of motor function indicators before and after intervention (mean ± SD, *n* = 19).

Test item	Pre-intervention	Post-intervention	Improvement (%)	SD	*p*	CV%	Cohen’s *d*	Effect size
Grip strength (right hand) (kg)	20.1 ± 5.6	23.1 ± 5.9	14.9%	4.36	*p* < 0.01	27.9%	0.54	Medium
1-min rope skipping (times)	85 ± 26	115 ± 30	35.3%	43.59	P < 0.01	30.6%	1.15	Large
1-min sit-ups (times)	22 ± 8	30 ± 9	36.4%	15.85	*p* < 0.05	36.4%	1.00	Large
Sit-and-reach (cm)	8.2 ± 4.5	11.2 ± 4.8	36.6%	6.08	P < 0.05	54.9%	0.67	Medium

### 3.4 Changes in eight-direction postural control

After the intervention, improvements were observed in all eight directions of postural control (Figure 3). Both endpoint excursion (EPE) and maximum excursion (MXE) significantly increased in all directions. Reaction time (RT) was significantly reduced in four directions: forward (F), left-back (LB), left (L), and right (R) (all *p* < 0.05), indicating that neural response efficiency improved preferentially in these directions. Directional control (DCL) showed a statistically significant improvement only in the left (L) direction (*p* < 0.05).

**Figure 3:**
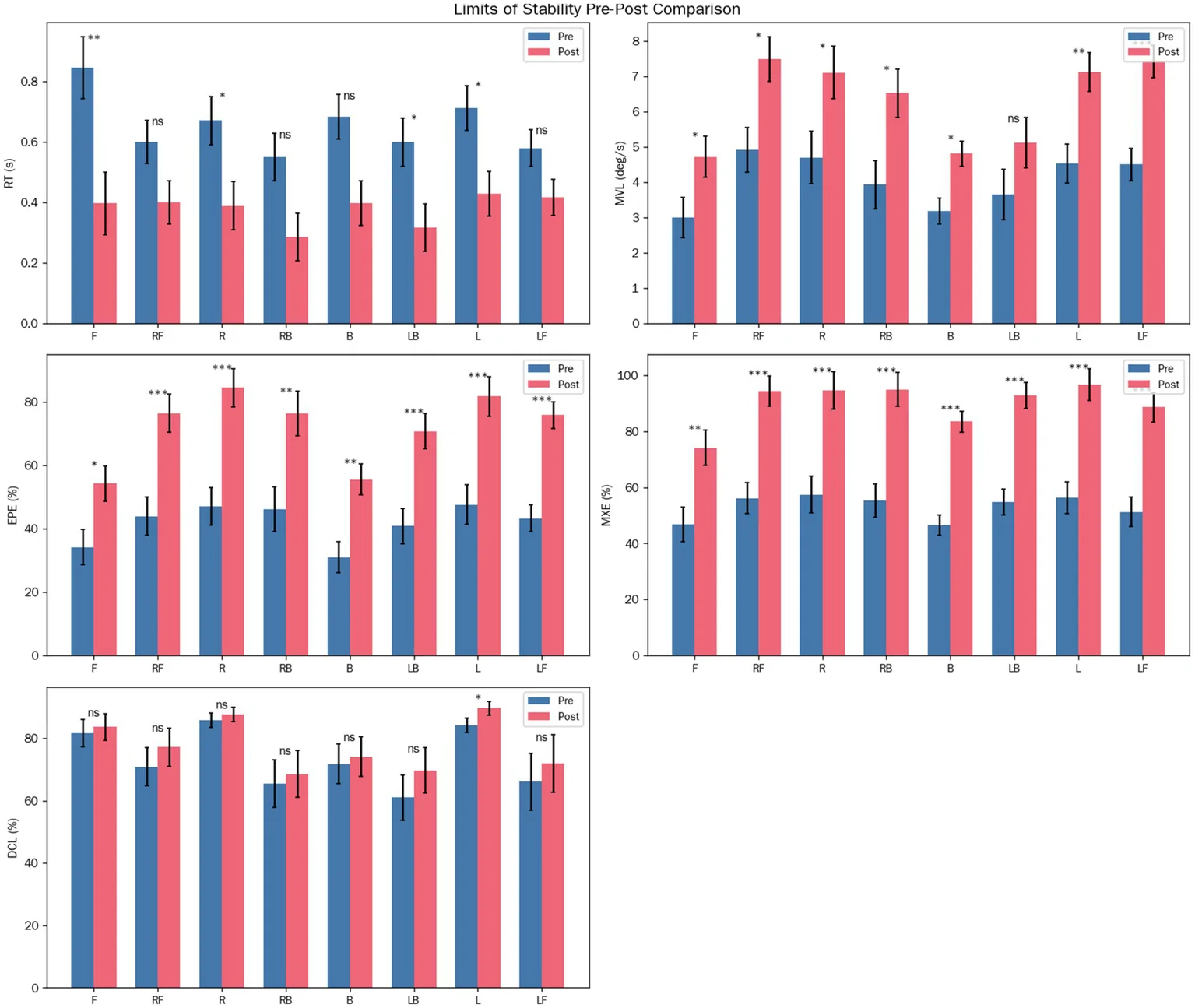
Comparison of eight-direction limits of stability test results before and after intervention (mean ± SD, *n* = 19). RT, reaction time; MVL, movement velocity; EPE, endpoint excursion; MXE, maximum excursion; DCL, directional control.

### 3.5 Sensory organization test results

As shown in Table 4, the mean balance percentages in all SOT conditions were generally higher after intervention than before. Significant improvements were observed for SOT-1, SOT-2, SOT-4, and SOT-5. The improvements were more pronounced under more challenging conditions. For SOT-4 (sway-referenced platform, fixed visual surround), the mean balance percentage increased from 60.6 ± 18.9 to 76.7 ± 10.8 (*p* = 0.005, Cohen’s *d* = 1.05, Δ = 26.7%). For SOT-5 (eyes closed, sway-referenced platform), it increased from 48.9 ± 18.0 to 59.7 ± 19.2 (*p* = 0.01, Cohen’s *d* = 0.58, Δ = 22.3%). Although SOT-3 and SOT-6 showed mean improvements, these did not reach statistical significance (*p* = 0.14 and 0.11), suggesting that the improvement trends were insufficient to draw firm statistical conclusions given the current sample size. The maximum balance percentage results were generally consistent with the average values. SOT-1, SOT-2, SOT-4, and SOT-5 showed significant improvements, with SOT-2 and SOT-4 achieving large effect sizes (Cohen’s *d* = 1.01 and 1.04), indicating substantial improvements in the participants’ best performance as well. However, the maximum changes for SOT-3 and SOT-6 did not reach statistical significance (*p* = 0.22 and 0.25).

**Table 4:** Comparison of sensory organization test results before and after intervention (*n* = 19).

Balance/Condition	Average (three trials)					Max				
	Pre-intervention	Post-intervention	*p*	Cohen’s *d*	Δ%	Pre-intervention	Post-intervention	*p*	Cohen’s *d*	Δ%
SOT-1	88.7 ± 7.8	94.0 ± 5.7	*p* < 0.01	0.78	6.0	91.8 ± 5.1	95.9 ± 4.5	*p* < 0.01	0.86	4.6
SOT-2	84.0 ± 7.9	89.6 ± 7.0	*p* < 0.01	0.75	6.7	87.2 ± 7.3	93.8 ± 5.7	*p* < 0.01	1.01	7.6
SOT-3	73.9 ± 11.7	79.9 ± 10.4	0.14	0.54	8.1	85.2 ± 7.0	87.9 ± 5.5	0.22	0.43	3.2
SOT-4	60.6 ± 18.9	76.7 ± 10.8	*p* < 0.01	1.05	26.7	75.9 ± 16.5	88.5 ± 5.0	*p* < 0.01	1.04	16.7
SOT-5	48.9 ± 18.0	59.7 ± 19.2	*p* < 0.01	0.58	22.3	60.8 ± 19.5	70.0 ± 19.9	*p* < 0.05	0.47	15.2
SOT-6	47.4 ± 22.1	56.2 ± 21.2	0.11	0.41	18.6	61.5 ± 24.5	69.1 ± 23.8	0.25	0.31	12.3

As shown in Figure 4, the SOT composite balance score increased significantly by 24.15% after the intervention (*p* < 0.001, Cohen’s *d* = 1.28).

**Figure 4:**
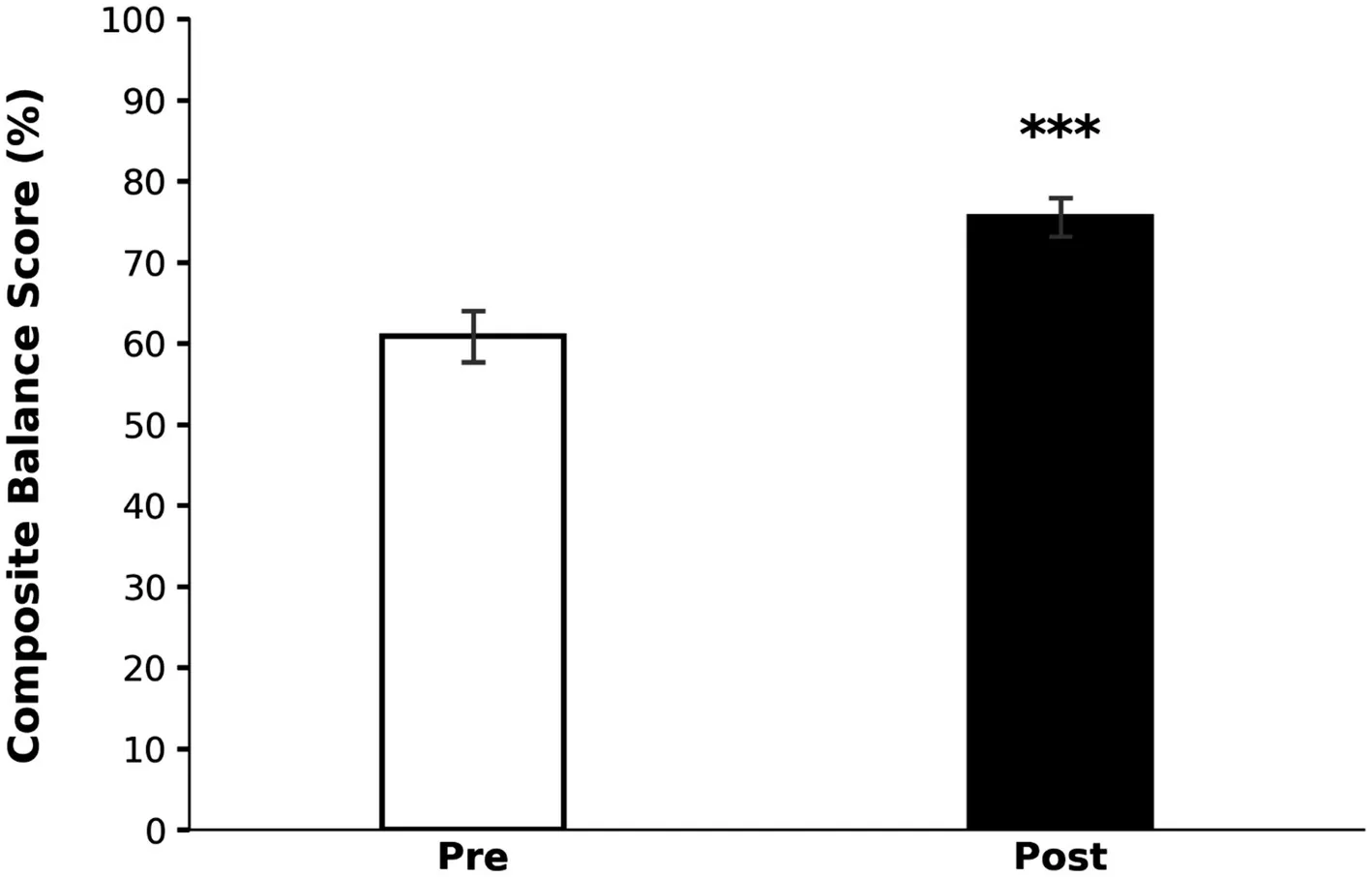
SOT composite balance score.

At the sensory integration level (Figure 5), the SOT somatosensory ratio was 0.947 before and 0.953 after intervention, representing a 0.6% change, with no significant difference (*p* = 0.915, Cohen’s *d* = 0.04). Paired-sample *t*-test based on individual ratios revealed no significant difference before and after intervention. This suggests that proprioceptive function was already relatively efficient before intervention and remained stable. The visual ratio increased from 0.683 to 0.816, a 19.5% increase (*p* < 0.05, Cohen’s *d* = 0.80). The vestibular ratio increased from 0.551 to 0.635, a 15.3% increase, which approached but did not reach statistical significance (*p* = 0.054, Cohen’s *d* = 0.43). These results indicate that the improvement in postural control was primarily driven by enhanced visual information utilization, while vestibular function also showed a positive trend toward improvement.

**Figure 5:**
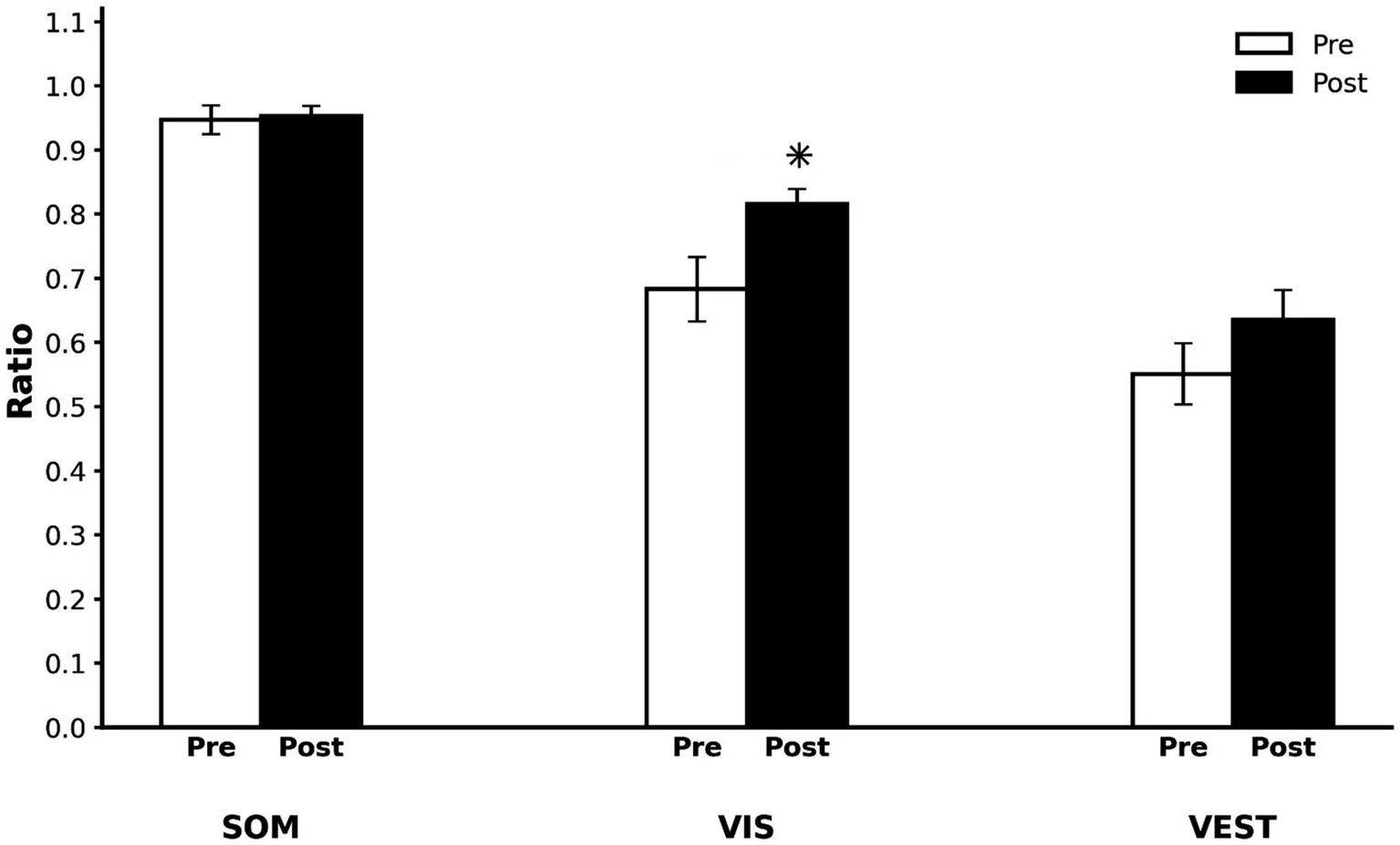
Changes in SOT sensory ratios pre- and post-intervention. The SOT’s somatosensory ratio = average (SOT-2)/average (SOT-1). The SOT’s visual ratio = average (SOT-4)/average (SOT-1). The SOT’s vestibular ratio = average (SOT-5)/average (SOT-1).

## 4 Discussion

This study was a single-arm pilot trial; therefore, all observed improvements should be interpreted as preliminary signals rather than definitive evidence of intervention efficacy, and the conclusions require confirmation in future randomized controlled trials. Within this context, we observed multidimensional improvement signals in children with obesity following the AI-assisted, deficit-oriented exercise intervention: significant reductions in BMI and body fat mass (*p* < 0.001) with preserved skeletal muscle mass; significant improvements in motor function, including grip strength, jump rope, sit-ups, and sit-and-reach (*p* < 0.01 to *p* < 0.05); widespread improvements in eight-directional postural control (EPE, MXE) and reduced reaction times in selected directions; and significant improvements in SOT-4, SOT-5, visual ratio, and composite balance scores (*p* < 0.05 to *p* < 0.001). Because dietary intake was not quantitatively monitored in this study, the improvements in body composition should be interpreted as a combined effect primarily driven by the exercise intervention and potentially synergized with dietary habits, rather than being attributable to exercise alone.

The “fat loss with muscle preservation” effect observed in this study is consistent with the meta-analyses by Liu et al. (23) and Huang et al. (24), both of which confirmed the synergistic benefits of combined aerobic and resistance training. However, whereas previous studies largely used fixed protocols, the present study achieved individualized, dynamic load adjustments through the AI system, representing a substantial improvement over traditional models. Regarding postural control, Colné et al. (25) reported that obese adolescents had significantly lower forward and backward tilt abilities than their normal-weight peers. This finding aligns precisely with the core logic of the “functional deficit-oriented” approach in our system: upon detecting weakness in specific directions, the system increases the training load for eccentric control of the relevant muscle groups, enabling targeted enhancement. Directional control improvement was observed only in the L direction (*p* < 0.05), which may be attributable to the fact that most children were right-side dominant, with relatively weaker left-side control; targeted training may have effectively corrected this strategic asymmetry. Güngörenler et al. (26) demonstrated that core stabilization training improves balance in obese children. Building on this, the present study further clarifies the directional specificity and potential neural mechanisms underlying these improvements. Compared with the remote app-based intervention by de Freitas et al. (13) the “AI prescription + on-site coach implementation” model in this study achieved 100% attendance, far exceeding the adherence levels of remote interventions, underscoring the critical importance of on-site supervision for pediatric populations. To address the clinical characteristics of obese children—reduced exercise tolerance and low motivation (27) —this model establishes a clear division of labor between the AI and the on-site coach: the AI generates the prescription and allocates training weights to areas of weakness, while the coach delivers on-site guidance and provides feedback, enabling the system to adjust training difficulty accordingly. This closed-loop mechanism ensures that children consistently train within a “challenging yet achievable” zone, which not only sustains long-term participation through repeated success experiences (28) but also balances safety and adherence through real-time intensity regulation.

Notably, the SOT results in this study reveal potential mechanisms by which the intervention improved sensory integration function. A recent large-sample cross-sectional study using the NeuroCom system confirmed that obese children exhibit a balance pattern relying more on hip strategies under SOT-5 (platform sway + eyes closed), with significantly worse endpoint excursion (EPE), indicating clear deficits under conditions demanding greater reliance on vestibular information (29). Building on this evidence, the present study found that the 12-week exercise intervention improved balance performance under these challenging conditions: SOT-4 (platform sway, fixed visual surround) increased from 60.6 to 76.7%, and SOT-5 (eyes closed, platform sway) increased from 48.9 to 59.7%. This finding provides direct empirical support for the reversibility of postural control deficits in children with obesity, suggesting that targeted exercise training can effectively compensate for obesity-related sensory integration deficits. Further analysis of sensory ratios revealed that the visual ratio increased significantly by 19.5%, the vestibular ratio increased by 15.3% and approached significance, whereas the somatosensory ratio remained stable at a high level. This pattern has important clinical implications: proprioceptive function in obese children may not be severely impaired; instead, postural control deficits may primarily stem from insufficient or inefficient utilization of visual and vestibular information. Through 12 weeks of multi-directional center-of-gravity control and unstable surface training, the central nervous system’s ability to dynamically adjust sensory weighting in response to multisensory conflicts—that is, “sensory reweighting” efficiency—was specifically improved, particularly through the optimization of visual and vestibular pathways. This mechanistic finding offers a new sensory integration perspective for understanding how exercise interventions improve postural control in obese children and suggests that future intervention protocols should prioritize visual-vestibular integration training.

Based on these findings, we propose the following practical recommendations. First, before prescribing exercise regimens for children with obesity, clinicians should incorporate an eight-directional postural control assessment into routine screening to identify individual functional weaknesses. Second, intervention programs should shift from a purely “weight reduction” orientation toward a synergistic “fat loss with muscle preservation and functional remodeling” approach, with special emphasis on eccentric control training of the posterior chain. Where resources permit, we recommend adopting a hybrid model of “AI-driven decision-making combined with human supervision” to enhance prescription precision and intervention adherence. Finally, community and school health programs could adapt this model by using intelligent systems for large-scale initial assessments and prescription generation, followed by group supervision led by physical education teachers or community health coaches, thereby optimizing both cost-effectiveness and outcomes.

### 4.1 Clinical implementation notes

First, this intervention model is suitable for children aged 6–15 years with simple obesity; it is not applicable to those with pathological obesity or severe contraindications to exercise. Second, implementation requires coaches who have undergone standardized training, and the AI system’s rule base should be updated regularly to maintain evidence-based currency. Third, if a child experiences sustained RPE ≥ 17 (“very hard”) or joint pain during training, the session should be terminated immediately, and the prescription should be reassessed.

### 4.2 Limitations

This study has several limitations. First, regarding dietary control, only verbal instructions were given to participating families to maintain their usual dietary habits; no quantitative dietary monitoring (e.g., food diaries, 24-h recalls) was performed. Therefore, although the intervention was exercise-based, the reduction in body fat may have been influenced by both exercise and potential dietary changes; thus, the fat loss cannot be attributed solely to exercise. Second, due to the small sample size and the single-group pre-post design without a parallel control group, the generalizability of the findings and the strength of causal inferences are limited. Third, multiple paired t-tests were performed on secondary outcomes without correction for multiple comparisons, which may have increased the risk of Type I errors (false positives). Therefore, the observed signals of improvement in secondary outcomes require confirmation in future, larger-sample confirmatory trials with a pre-registered analysis plan. Fourth, the 12-week intervention period was insufficient to assess long-term sustainability and weight rebound. Finally, the AI system’s prescription rules were based on a specific expert knowledge base, and their generalizability across diverse populations and settings remains to be further validated.

## 5 Conclusion

In summary, this single-arm pilot study provides preliminary feasibility evidence and improvement signals for the application of an AI-assisted, deficit-oriented exercise intervention in children with obesity. The findings suggest that interventions for childhood obesity should not be limited to weight reduction but may also positively influence postural control strategy optimization and neuromuscular function remodeling. The deficit-oriented exercise intervention, supported by an AI system that dynamically adjusts training resources to target individual weaknesses, demonstrates preliminary application potential and offers a new approach for early functional screening and precision exercise management in pediatric obesity. However, the efficacy of this model requires further confirmation in subsequent confirmatory trials. Future research should expand sample sizes, adopt randomized controlled designs, include objective dietary records, and ensure trial registration to validate the effectiveness and mechanisms of this intervention model.

## Data Availability

The data analyzed in this study is subject to the following licenses/restrictions: The data presented in this study are available on reasonable request from the corresponding author. The data are not publicly available due to privacy or ethical restrictions related to the participation of minors. Requests to access these datasets should be directed to caolixin, cao0326xin@163.com.
